# Molecular cloning, expression, and functional analysis of the chitin synthase 1 gene and its two alternative splicing variants in the white-backed planthopper, *Sogatella furcifera* (Hemiptera: Delphacidae)

**DOI:** 10.1038/s41598-018-37488-5

**Published:** 2019-01-31

**Authors:** Zhao Wang, Hong Yang, Cao Zhou, Wen-Jia Yang, Dao-Chao Jin, Gui-Yun Long

**Affiliations:** 1 Institute of Entomology, Guizhou University, Provincial Key Laboratory for Agricultural Pest Management of Mountainous Regions, Guiyang, 550025 P. R. China; 2 College of Environment and Life Sciences, Kaili University, Kaili, 556011 P. R. China; 3 College of Tobacco Science of Guizhou University, Guiyang, 550025 P. R. China; 4 Guizhou Provincial Key Laboratory for Rare Animal and Economic Insects of the Mountainous Region, College of Biology and Environmental Engineering, Guiyang University, Guiyang, 550005 P. R. China

## Abstract

Chitin synthase is responsible for chitin synthesis in the cuticles and cuticular linings of other tissues in insects. We cloned two alternative splicing variants of the chitin synthase 1 gene (*SfCHS1*) from the white-backed planthopper, *Sogatella furcifera*. The full-length cDNA of the two variants (*SfCHS1a* and *SfCHS1b*) consists of 6408 bp, contains a 4719-bp open reading frame encoding 1572 amino acids, and has 5′ and 3′ non-coding regions of 283 and 1406 bp, respectively. The two splicing variants occur at the same position in the cDNA sequence between base pairs 4115 and 4291, and consist of 177 nucleotides that encode 59 amino acids but show 74.6% identity at the amino acid level. Analysis in different developmental stages showed that expression of *SfCHS1* and *SfCHS1a* were highest just after molting, whereas *SfCHS1b* reached its highest expression level 2 days after molting. Further, *SfCHS1* and *SfCHS1a* were mainly expressed in the integument, whereas *SfCHS1b* was predominately expressed in the gut and fat body. RNAi-based gene silencing inhibited transcript levels of the corresponding mRNAs in *S*. *furcifera* nymphs injected with double-stranded RNA of *SfCHS1*, *SfCHS1a*, and *SfCHS1b*, resulted in malformed phenotypes, and killed most of the treated nymphs. Our results indicate that *SfCHS1* may be a potential target gene for RNAi-based *S*. *furcifera* control.

## Introduction

Chitin, a linear homopolymer of *N*-acetylglucosamines (GlcNAc) linked by β-1,4 glycosidic bonds, is the second most abundant biological polysaccharide in nature after cellulose^1,2^. It is widely distributed in fungi, sponges, nematodes, mollusks, arthropods, fishes, amphibians and some algae^2–5^. In insects, chitin has been verified as a crucial structural constituent of the cuticle, alimentary canal, tracheal system, genital ducts, and ducts of various dermal glands^6^, and plays a major role in maintaining body shape and protecting from external mechanical disruption^7,8^. To allow growth and development, insects must periodically digest their old cuticle and produce a new and looser one during molting^2^. Chitin synthase (CHS; EC 2.4.1.16) is a vital enzyme involved in the final step of the chitin synthesis pathway. CHS is a highly conserved enzyme found in all chitin-containing organisms^9,10^. Insect CHSs are large transmembrane proteins that belong to family 2 glycosyltransferases^2^. To date, CHSs have been cloned and sequenced in various insect species from different orders, including Coleoptera^11,12^, Lepidoptera^13–15^, Orthoptera^16,17^, Hemiptera^10,18–20^, and Diptera^21–23^. On the basis of their sequence similarity, distribution, and physiological functions, insect chitin synthases are categorized into two types: CHS1 and CHS2^24^. CHS1 is primarily responsible for the formation of chitin utilized in the cuticle and tracheae, as well as in the linings of the foregut and hindgut, whereas CHS2 is dedicated to chitin synthesis in the peritrophic membrane (PM) of the midgut^25^. However, some reports have pointed out that hemipteran insects such as *Aphis glycines*, *Rhodnius prolixus* and *Nilaparvata lugens* lack PM. Instead, these insects have the perimicrovillar membrane (PMM), a similar structure to PM that covers the microvilli of midgut. This structure is important for digesting and protecting against attacks from microorganisms^10,19,26,27^. Additionally, it has also been reported that insect *CHS1* contains alternative exon which results in the production of two alternative splicing variants, *CHS1a* and *CHS1b*. The two variants are different in a 177 bp region that encode 59 amino acid residues in all insects examined so far^6,28^. Nevertheless, alternative splicing variants have not been reported for the gene encoding CHS2^16,19,23^. To date, the functions of the *CHS* genes have been extensively investigated using RNA interference (RNAi) in both holometabolous and hemimetabolous insects such as *Tribolium castaneum*^29,30^, *Anthonomus grandis*^31^, *Spodoptera exigua*^32^, *Bactrocera dorsalis*^23^, *Drosophila melanogaster*^33,34^, *Locusta migratoria*^16,17^, *Laodelphax striatellus* and *N*. *lugens*^19^. These results have indicated that *CHS* genes are essential for survival, ecdysis, fecundity, and egg hatching. Moreover, in *D*. *melanogaster*, histological analysis of mutants for the *CS-1* gene (also called *krotzkopf verkehrt*) indicated that chitin formation and differentiation are crucial for procuticle integrity and for attachment of cuticle to the epidermal cells^35^. To sum up, chitin biosynthesis is pivotal for insect growth and development, and the CHS enzymes participating in chitin biosynthesis are promising targets for the design of novel strategies for the control of insect pests.

The white-backed planthopper, *Sogatella furcifera* (Horváth), is a serious insect pest that affects rice crops in some Asia-Pacific countries. In China, the outbreak frequency of *S*. *furcifera* has been increasing in recent years^36^. This pest causes severe losses in rice production by sucking, ovipositing, and transmitting viruses^37^. Because of its high fecundity, long-distance migration, and its quick development of resistance against pesticides, it is difficult to control this pest using traditional chemicals. Previous studies have demonstrated that RNAi technology has considerable potential in the control of serious pests by silencing vital genes^38^; for example, double-stranded RNA (dsRNA) can be absorbed orally by *N*. *lugens* and lead to reduced expression levels of target genes^39,40^. Thus, transgenic rice that expresses dsRNAs corresponding to vital hemipteran pest genes could be used for the control of these insect pests^40^. Accordingly, it is also important to identify a lethal gene(s) for developing an RNAi-based technique that can be used in the control of the hemipteran pest *S*. *furcifera*.

In this study, we cloned and characterized a full-length cDNA encoding chitin synthase 1 (*SfCHS1*) from *S*. *furcifera*, identified two alternative splicing variants (*SfCHS1a* and *SfCHS1b*) of *SfCHS1*, and analyzed the expression patterns of *SfCHS1* and the two alternative variants at different developmental stages and in different tissues. Moreover, we demonstrate that dsRNA-mediated gene-specific silencing resulted in a strong reduction in the transcript levels of the target genes and insect survival rates. We also describe lethal phenotypes of *S*. *furcifera* induced by target gene silencing.

## Results

### Identification and characterization of *SfCHS1*

**Figure 1:**
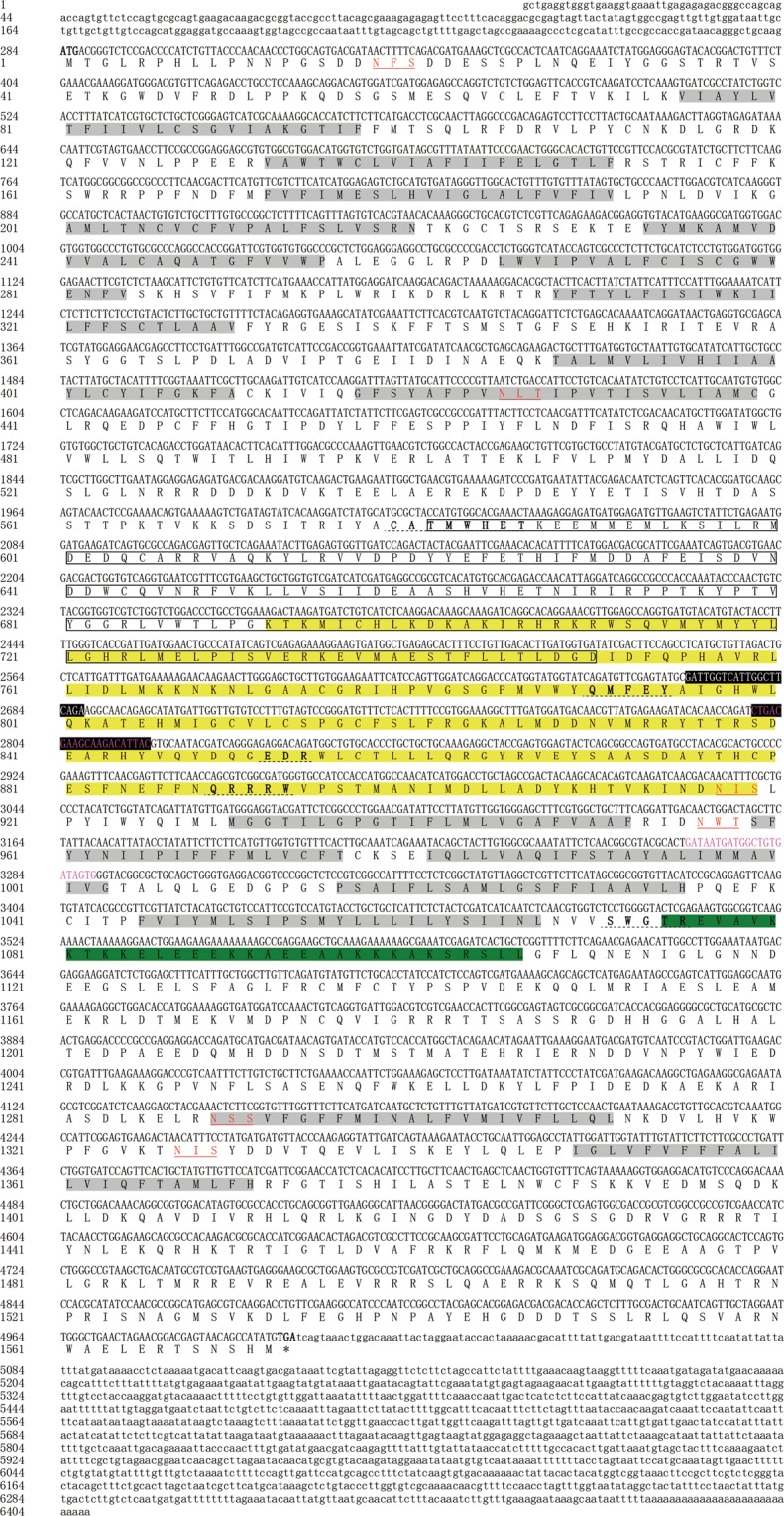
Full-length nucleotide and deduced amino acid sequences of *SfCHS1a* cDNA from *S*. *furcifera* (KY350143). The start codon (ATG) is highlighted in bold and the stop codon (TGA) in bold with asterisk. The 16 transmembrane helix regions predicted by TMHMM Server v2.0 are indicated in gray. The ligand-binding site predicted by 3DLigandSite is boxed, and the putative catalytic domain is highlighted in yellow. The six putative *N*-glycosylation sites predicted by NetNGlyc 1.0 Server are underlined in red. The chitin synthase signature motifs are highlighted in bold italic with a dotted line. Predicted coiled-coil regions are indicated by a green background. The primers of *SfCHS1* for qPCR analysis are indicated by a black background, and the primers for dsRNA synthesis are highlighted in pink.

The full-length cDNA sequence of *SfCHS1* was obtained by multiple PCR amplifications and RACE. The full-length nucleotide and deduced amino acid sequences of *SfCHS1* are shown in Fig. 1. The complete cDNA sequence of *SfCHS1* is 6,408 bp in size. The ORF of *SfCHS1* is 4,719 bp long and encodes a protein of 1,572 amino acid residues with a predicted molecular weight of 180.6 kDa and a pI of 6.72. The *SfCHS1* cDNA includes a 5′ non-coding region of 283 bp and a 3′ non-coding region of 1,406 bp.

On the basis of the deduced amino acid sequence, 16 transmembrane helices (TMHs) were predicted using the TMHMM Server v.2.0, suggesting that SfCHS1 is a membrane-associated protein. Similar to other known insect CHS proteins, SfCHS1 has an N-terminal domain (domain A) containing nine TMHs; a central domain (domain B) that contains two signature motifs, EDR (852–854) and QRRRW (889–893), and two other motifs that are highly conserved in insect chitin synthases, CATMWHET (579–586) and QMFEY (790–794)^41^; and a C-terminal domain (domain C) that contains seven TMHs and another signature motif SWGTR (1071–1075) that may play a role in chitin translocation^2,42^. Using the 3DLigandSite Server^43^, a ligand-binding site was identified in the amino acid region 581–750, and a putative catalytic domain at position 579–900 was predicted using the SMART program. The Paircoil program identified a coiled-coil region following transmembrane helix five of the C domain. In addition, six possible N-glycosylation sites were predicted using the NetNGlyc 1.0 Server, suggesting that the SfCHS1 protein may be glycosylated. However, analysis of deduced amino acid sequences using the SignalP 4.1 Server did not identify a signal peptide.

### Comparative analysis of alternative splicing exons of *SfCHS1*

**Figure 2:**
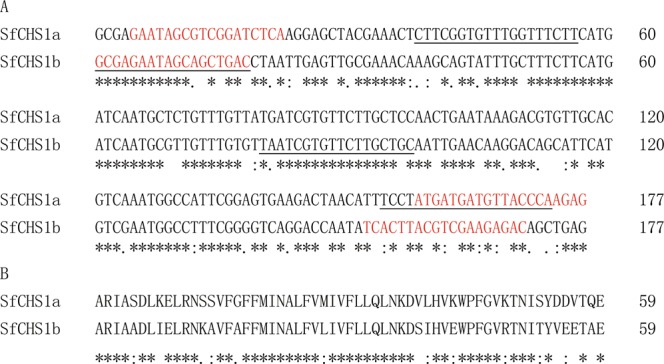
Comparative analysis of two alternative splicing variants of *SfCHS1* in *S*. *furcifera*. Alignment of nucleotide (**A**) and deduced amino acid (**B**) sequences of *SfCHS1* alternative exon-a and exon-b using Clustal Omega software. Symbols below the alignments show identical (*), highly conserved (:), and conserved residues (.). The primers of *SfCHS1a* and *SfCHS1b* for qPCR analysis are underlined. The primers for dsRNA synthesis are highlighted in red.

Analysis of the *SfCHS1* cDNA sequence revealed two alternative splicing variants, named *SfCHS1a* and *SfCHS1b* (deposited in GenBank with accession numbers KY350143 and KY350144). The alternative exons are found in the same region (4115–4291) of the *SfCHS1* cDNA (Fig. 1), and have 177 nucleotides that encode 59 amimo acid residues (Fig. 2). Alignment of the deduced amino acid sequences indicated that the identity between SfCHS1a and SfCHS1b is 74.6%. Each exon codes for a highly conserved transmembrane helix, and the flanking sequences consist of an intracellular and an extracellular domain, respectively^24,44^.

### Sequence alignment and phylogenetic analysis

Multiple sequence alignment of CHS1 proteins indicated a high degree of amino acid sequence homology among different insect species. For instance, the SfCHS1 protein shows 98% and 97% identity with that from the hemipteran *L*. *striatellus* (LsCHS1, AFC61179) and *N*. *lugens* (NlCHS1, AFC61181), respectively. It also shares identities of 81%, 73%, 71%, and 70% with the chitin synthases of *Anasa tristis* (AtCHS1, AFM38193), *A*. *glycines* (AgCHS1, AFJ00066), *Cnaphalocrocis medinalis* (CmCHS1, AJG44538), and *T*. *castaneum* (TcCHS1, NP_001034491), respectively.

**Figure 3:**
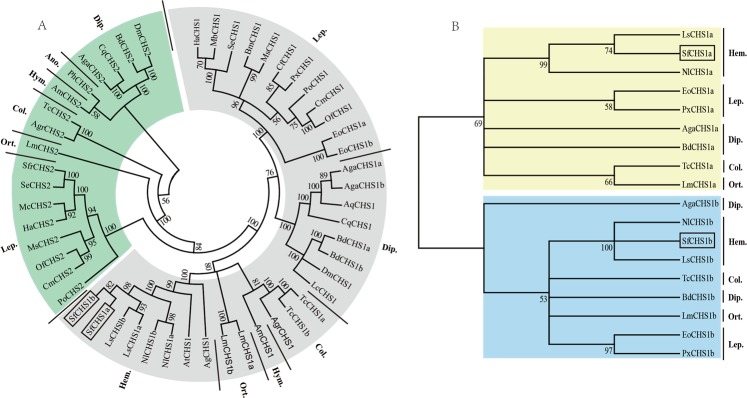
Phylogenetic trees of the known insect chitin synthases and alternative exons. (**A**) Tree of the known insect chitin synthases. (**B**) Tree of the alternative exons of insect CHS1s. The trees were constructed using MEGA 6.06 with the neighbor joining (NJ) method. Bootstrap analyses of 1000 replications were carried out and bootstrap values are shown next to the branches. The following insect chitin synthase sequences were used: *Anasa tristis* (At), *Aphis glycines* (Ag), *Laodelphax striatellus* (Ls), *Nilaparvata lugens* (Nl), *Bombyx mori* (Bm), *Choristoneura fumiferana* (Cf), *Cnaphalocrocis medinalis* (Cm), *Ectropis obliqua* (Eo), *Helicoverpa armigera* (Ha), *Mamestra brassicae* (Mb), *Mamestra configurata* (Mc), *Manduca sexta* (Ms), *Ostrinia furnacalis* (Of), *Phthorimaea operculella* (Po), *Plutella xylostella* (Px), *Spodoptera exigua* (Se), *Spodoptera frugiperda* (Sfr), *Apis mellifera* (Am), *Pediculus humanus corporis* (Ph), *Anthonomus grandis* (Agr), *Tribolium castaneum* (Tc), *Anopheles gambiae* (Aga), *Anopheles quadrimaculatus* (Aq), *Bactrocera dorsalis* (Bd), *Culex quinquefasciatus* (Cq), *Drosophila melanogaster* (Dm), *Lucilia cuprina* (Lc), *Locusta migratoria manilensis* (Lm). Lep.: Lepidoptera, Dip.: Diptera, Col.: Coleoptera, Hym.: Hymenoptera, Ort.: Orthoptera, Hem.: Hemiptera, Ano.: Anoplura. The accession numbers for various chitin synthases used in the phylogenetic analysis are provided in the Materials and methods section.

On the basis of the amino acid sequences of known insect CHSs, a phylogenetic tree was constructed using MEGA 6.06 based on the neighbor-joining method. The result indicated that the *CHS1* and *CHS2* genes originated from one ancestral gene and are closely related, but they clearly grouped into two different phylogenic branches (Fig. 3). The result is consistent with the findings of the previous studies^1,2,19,26^. Further, all hemipteran chitin synthases appeared to have a common ancestor in the lineage as indicated by the high bootstrap values (82~100), but they seemed to have lost the *CHS2* gene during subsequent evolution. The chitin synthase from *S*. *furcifera*, *SfCHS1*, is clustered into the *CHS1* family in the tree, and the identity of *SfCHS1* to *CHS1s* was markedly higher than identity to *CHS2s* from other insects (Fig. 3A). Moreover, the two splicing variants, *SfCHS1a* and *SfCHS1b*, grouped into two different phylogenetic classes (Fig. 3B).

### Developmental- and tissue-specific expression of *SfCHS1* and its two alternative splicing variants

**Figure 4:**
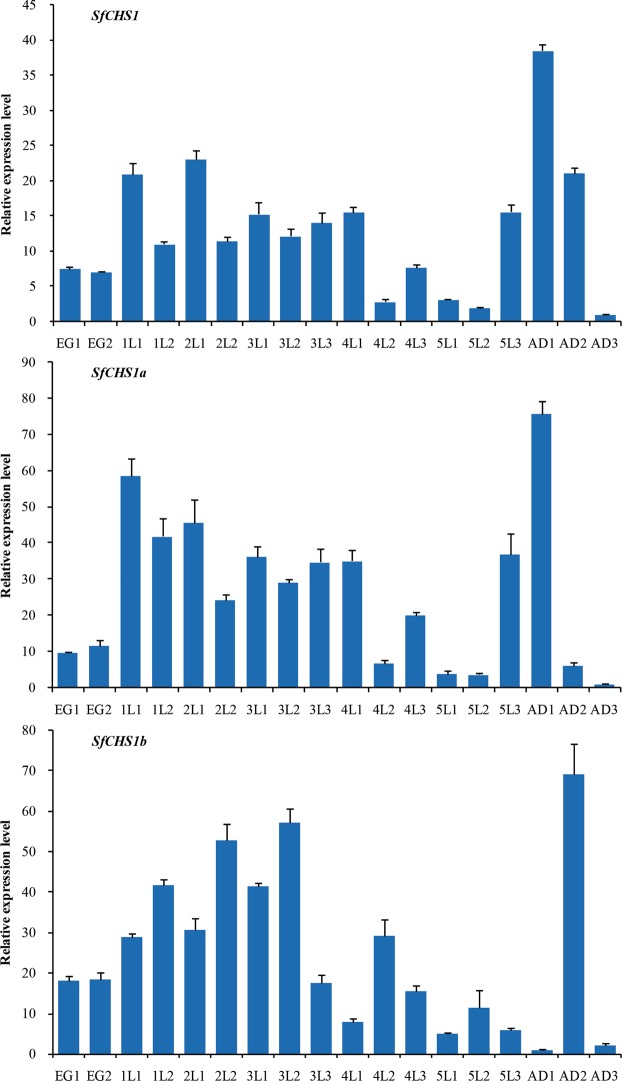
Relative expression levels of *SfCHS1* and its two alternative splicing variants in different developmental stages of *S*. *furcifera*. Expression levels at 18 different time points in eggs, nymphs (from first-instar to fifth-instar nymphs), and adults were determined by qPCR. The *S*. *furcifera 18S rRNA* was used as an internal reference gene. The relative expression was calculated based on the value of the lowest expression which was arbitrarily set to 1. Data are means ± *SE* of three biological replications. The age in days of the insects is indicated, e.g., EG1, first day of eggs; lL1, first day of first-instar nymphs; AD1, first day of adults.

qPCR was used to analyze the expression profiles of *SfCHS1* and its two alternative splicing variants at different developmental stages (Fig. 4). The results revealed that *SfCHS1* and its alternative variants were constitutively expressed in the 18 examined developmental stages. The relative expression levels of *SfCHS1* were higher just after each molting and reached a peak 1 day after eclosion. Specifically, the lowest expression levels for *SfCHS1* were observed in third-day adults. For *SfCHS1a*, the expression patterns appeared to be similar to those of *SfCHS1*, but the relative transcript levels were lower in second-day adults. In contrast, *SfCHS1b* showed a different expression pattern to *SfCHS1* and/or *SfCHS1a*, with the highest expression level being recorded 2 days after each molt.

**Figure 5:**
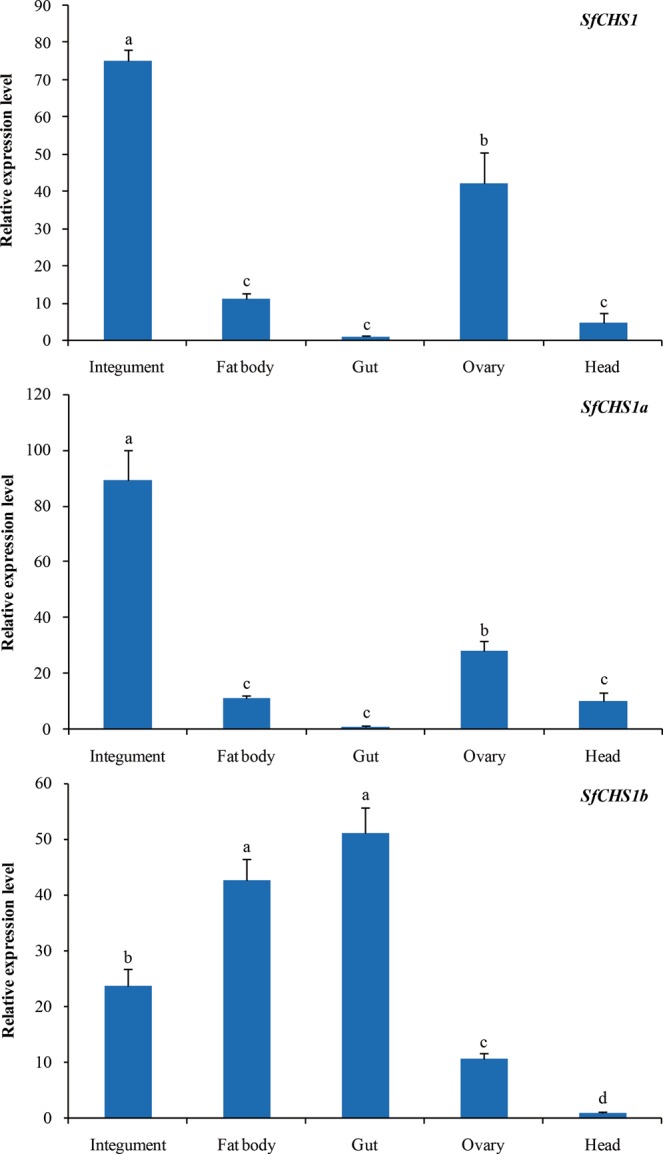
Expression profiles of *SfCHS1* and its two alternative splicing variants in different tissues of *S*. *furcifera*. The *S*. *furcifera 18S rRNA* was used as an internal reference gene. The relative expression was calculated based on the value of the lowest expression which was arbitrarily set to 1. Data are means ± *SE* of three biological replications. Different lower-case letters above the bars indicate significant differences (*P* < 0.05, Duncan’s multiple range test in One-way ANOVA).

To investigate where *SfCHS1* and its two alternative splicing variants are expressed, five different tissues from the integument, fat body, gut, ovary, and head were dissected for a tissue-specific expression experiment (Fig. 5). The results showed that *SfCHS1* was mainly expressed in the integument, and that its expression was 75-, 11-, 42-, and 5-fold higher in the integument, fat body, ovary, and head than in the gut, respectively. *SfCHS1a* was also predominantly expressed in the integument, whereas *SfCHS1b* was primarily expressed in the gut and fat body.

### RNAi response induced by injection of dsRNA

**Figure 6:**
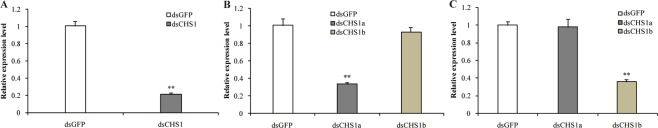
Relative transcript levels of *SfCHS1*, *SfCHS1a* and *SfCHS1b* after specific RNAi. (**A**) Transcript levels of *SfCHS1* of the fifth instar nymphs injected with dsGFP or dsSfCHS1. (**B**) Transcript levels of *SfCHS1a* of the fifth instar nymphs injected with dsGFP, dsSfCHS1a or dsSfCHS1b. (**C**) Transcript levels of *SfCHS1b* of the fifth instar nymphs injected with dsGFP, dsSfCHS1b or dsSfCHS1a. The *S*. *furcifera 18S rRNA* was used as an internal reference gene. Data are means ± *SE* of three biological replications. Significant differences between treatment and control are indicated with (**P < 0.01, t - test).

To verify whether RNAi is able to decrease target gene expression, sequence-specific dsRNAs for *SfCHS1*, *SfCHS1a*, and *SfCHS1b* were prepared *in vitro* and injected into first-day fifth-instar nymphs. Thereafter, qPCR was performed using total RNA isolated from dsRNA-injected insects as templates. The qPCR analysis indicated that the transcript levels of the target genes were markedly down-regulated at 72 h after dsRNA injection when compared with those of ds*GFP*-injected control insects (Fig. 6). More specifically, the expression of *SfCHS1* was reduced by approximately 79% in the ds*SfCHS1*-injected nymphs. After RNAi of the *SfCHS1a* gene, there was no decrease in the level of *SfCHS1b* mRNA, even though *SfCHS1a* expression showed a 67% decrease. Similarly, after RNAi of *SfCHS1b*, the transcript level of *SfCHS1b* was reduced by approximately 64%, whereas *SfCHS1a* expression did not appear to be affected. Consequently, we assumed the dsRNA-mediated silencing to be gene specific.

**Figure 7:**
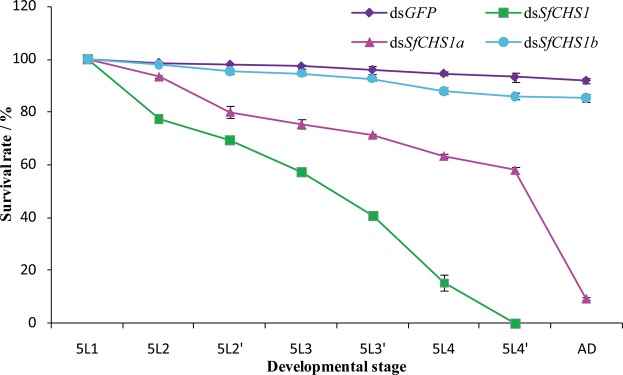
Survival rates after injection of dsRNA of *SfCHS1*, *SfCHS1a* and *SfCHS1b*. The survival rate of insects following the injection of dsRNAs on the first-day of fifth-instar nymphs. 100 ng dsRNA was injected into each nymph. The age in days of the insects is indicated, e.g., 5L1, first day of fifth-instar nymphs; 5L2 and 5L2′ represent the two 12 hours in 1 day; AD, adults. Data are mean ± *SE* from three biological replications with fifty insects in each group.

After successful silencing of *SfCHS1* and the two alternative splicing variants, mortality rates and lethal phenotypes of injected insects were recorded. It was clearly apparent that nymphs injected with 100 ng/head *SfCHS1* dsRNA could not shed their old cuticle, and were trapped within the exuviae, leading to 100% mortality (Fig. 7). Following *SfCHS1a* dsRNA injection, 42% of individuals died before reaching the adult stage. Nevertheless, 49% of individuals died after eclosion, among which 36% of nymphs were able to molt to become adults but exhibited a notably abnormal phenotype. Moreover, 13% failed to shed their appendages and eventually died (Fig. 7). Following *SfCHS1b* dsRNA injection, only 15% of nymphs died before eclosion, whereas 85% of individuals successfully underwent molting to become adults. In contrast, 92% of individuals in the ds*GFP*-injected control group survived and had a normal phenotype (Fig. 7).

**Figure 8:**
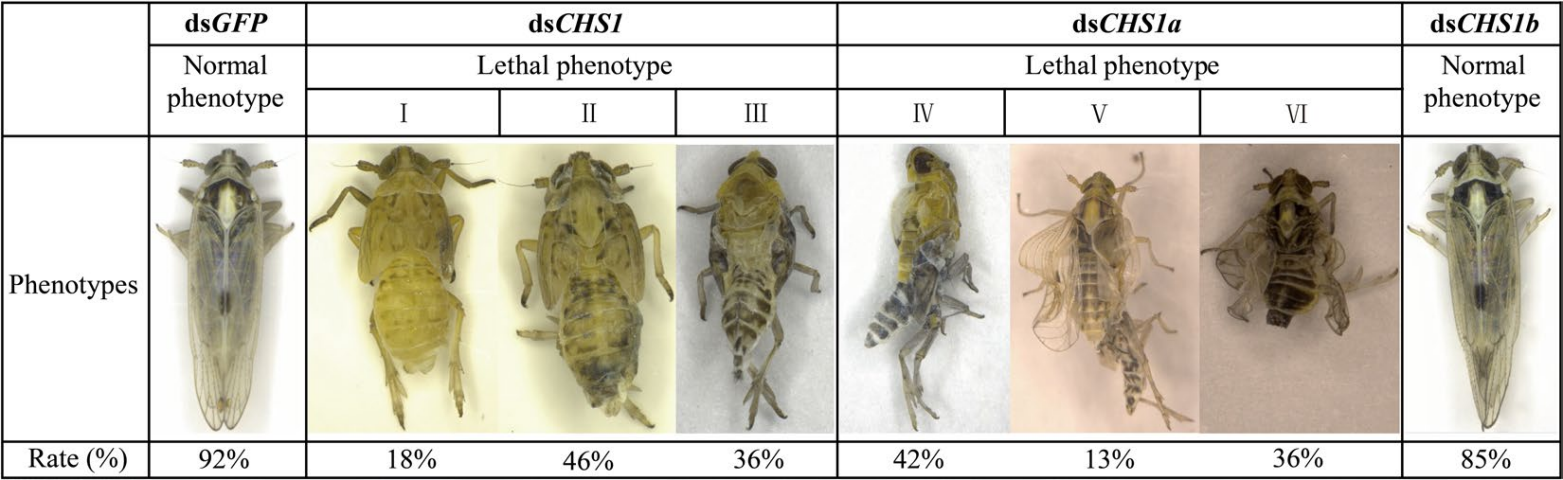
Representative phenotypes of *S*. *furcifera* after injection of *SfCHS1*, *SfCHS1a* and *SfCHS1b* dsRNA.

The fifth-instar nymphs of *S*. *furcifera* subjected to RNAi for the *SfCHS1* gene displayed several distinct phenotypes. When injected with dsRNA of *SfCHS1*, three abnormal phenotypes were observed, and the insects eventually died: shrunken abdomen that was smaller than that of normal nymphs (I); the old cuticle only slightly splitted open on the head and thorax (II); and the old cuticle cracked to certain level but the whole insect body was still encased (III) (Fig. 8). After injection with *SfCHS1a* dsRNA, three typical lethal phenotypes were present, which included: nymths partially shed their old cuticle but the old cuticle could not be completely detached from the body, particularly from the tail (IV); nymphs were able to molt and become adults, but the adults were unable to extricate their appendages (V); and nymphs molted successfully but the new cuticle was crimpled and the wings were malformed (VI) (Fig. 8). However, we found no obvious differences in visible phenotypes between individuals in the ds*SfCHS1b*- and ds*GFP*-injected groups (Fig. 8).

## Discussion

Chitin synthases play important roles in chitin biosynthesis during insect growth and development. It is known that most insects usually possess both *CHS1* and *CHS2*. *CHS1* is primarily expressed in the exoskeleton structures and is crucial for the synthesis of chitin required for the cuticle and tracheae, whereas *CHS2* is expressed in midgut epithelial cells for production of chitin in the PM^25^. In this study, we obtained the full-length cDNA encoding chitin synthase from the hemipteran *S*. *furcifera*. Alignment and phylogenetic analysis indicated that CHS from *S*. *furcifera* belongs to the CHS1 group. By searching of the genomes and transcriptomes of the hemipteran insects, it was demonstrated that these species seem to lost one of the two *CHS* genes during evolution, and only one *CHS* gene exists^18–20^. This result is probably associated with the fact that Hemiptera insects lack the PM^26^. Our result also indicated that the *SfCHS1* cDNA sequence is 6,408 bp in length and encodes a protein with a predicted pI of 6.72. The slightly acidic pI is conducive to its function in the cuticle. Similar to the CHS1 protein of other insects, SfCHS1 was predicted to be a 180.6-kDa membrane protein that contains 16 TMHs. The distribution and conserved number of these transmembrane segments in SfCHS1 allow the central catalytic domain (domain B) to face the cytoplasm, where the UDP-*N*-acetylglucosamine (UDP-GlcNAc) substrate is accessible. Its catalytic domain contains the highly conserved chitin synthase signature motifs CATMWHET, QMFEY, EDR, and QRRRW, which have been implicated to be essential for the catalytic mechanism^1,41,45^. Among the 16 TMHs, five are located immediately adjacent to the catalytic domain, forming a topological feature named the five-transmembrane span (5-TMS) region. This topology is found in all insect chitin synthases^18,19,23,24,46^. Consistent with other insect CHS1 proteins, SfCHS1 was predicted to include a conserved coiled-coil region immediately following the 5-TMS region, which is orientated toward the extracellular space and is a potential region for protein–protein oligomerization, or functions as a signal for vesicular trafficking^19,23,47–49^.

Alternative splicing plays a vital role in regulating gene function by expanding the diversity of expressed mRNA transcripts^46^. Many previous studies have demonstrated that alternative splicing appears to occur in the *CHS1* gene^1,46,50^. In the present study, we also detected the presence of two alternative splicing exons of 177 bp in *SfCHS1*. However, it is surprising that no alternative exons have been identified in the genome of the hemipteran insect *A*. *glycines*^18^. A similar absence of alternative exons has also been reported in the hemipteran *Toxoptera citricida*^20^ and thus it appears that alternative exons of the *CHS1* gene are present in *S*. *furcifera* but are absent in aphids. The relationship between the production and evolution of alternative splicing thus requires further investigation.

In the present study, we performed qPCR expression analysis of *SfCHS1* and its two alternative exons at different developmental stages in *S*. *furcifera*. Our results indicated that the expression of *SfCHS1* was periodically repeated at each molting cycle. The transcript level of *SfCHS1* peaked after molting, declined during each inter-molting phase and then increased again before the next molt, which may be associated with the requirement of chitin. Similar phenomena have also been observed for the transcript patterns of *CHS1* in *N*. *lugens*^19^, *Manduca sexta*^41^, *T*. *castaneum*^46^ and *Ostrinia furnacalis*^14^. Indeed, previous studies have shown that CHS1 is essential for eggshell formation and egg hatching in *T*. *castaneum*^30^, and that *CHS1a* mRNA expression plays a vital role in chitin synthesis of the serosal cuticle in *Aedes aegypti*^44^. In the current study, we also observed a relatively high expression of *SfCHS1* in *S*. *furcifera* eggs. These results indicate that constitutive expression of *SfCHS1* might be necessary in *S*. *furcifera*. Furthermore, the developmental expression patterns of *SfCHS1a* were similar to those of *SfCHS1*, but differed from those of *SfCHS1b*. Similar results were obtained by Wang *et al*.^19^ in *N*. *lugens* and Yang *et al*.^23^ in *B*. *dorsalis*. These results accordingly indicate that *SfCHS1a* and *SfCHS1b* probably play different roles in the biosynthesis of chitin during insect growth and development.

Further, the expression profiles of *SfCHS1* and its two alternative exons were also investigated in various tissues. The results showed that *SfCHS1* was predominately expressed in the integument, and ovary, with the highest levels of expression being observed in the integument. This is consistent with the fact that *CHS1* is responsible for chitin biosynthesis in the epidermis. However, *SfCHS1* was expressed at very low levels in the gut. Although the hemipteran insects lack PM, chitin was also detected in the lining of the gut of *Myzus persicae*^51^. The trace amounts of *SfCHS1* transcripts in the gut might be responsible for the chitin-containing structures. Additionally, the observed low expression of *SfCHS1* mRNA in the gut might be alternatively explained by the fact that the tracheae are tightly integrated into gut tissues and thus it is very difficult to completely remove these from the gut due to small size of the body^52^. The weaker expression of *CHS1* in the gut was also detected in *L*. *migratoria*^16^, *N*. *lugens*^19^ and *Plutella xylostella*^53^ and these were believed to be due to contamination from the tracheal tissues. Also, we had detected a relatively high level of expression in the ovary. Similar results have been observed in *Mythimna separata*^54^, where *MsCHS1* was highly expressed in the ovary. A previous study using the fluorescently labeled lectin technique had also documented that chitin was present in *A*. *aegypti* ovaries as well as in the eggs and egg shells^55^, suggesting the importance of *CHS1* gene in insect reproduction. A low expression of *SfCHS1* in *S*. *furcifera* head was also observed. Similar results have also been observed in *P*. *xylostella*^53^ and *Bombyx mori*^15^, where the *CHS1* gene was expressed in their head. Expression of *CHS1* is known to be integument-specific. Therefore, we speculated that expression in the head was probably due to the *CHS1* gene in the epidermis of the head. Moreover, we noted that the expression patterns of *SfCHS1a* were similar to those of *SfCHS1*, with the highest levels in the integument, whereas an exceedingly high expression of *SfCHS1b* was detected in the gut and fat body. However, a previous study on *Anopheles gambiae* has shown that *AgCHS1a* and *AgCHS1b* share the same transcript patterns and are expressed at considerable levels in the carcass (ie the insect body after its digestive canal is removed)^56^. Future work will be needed to address how *CHS1a* and *CHS1b* are involved in the physiological function of the various tissues in different insect species.

Gene silencing through dsRNA feeding and dsRNA injection has been successfully used for studying the functions of essential genes in hemipteran insects^10,19,20,39,57–60^. In the present study, to ascertain the functional difference among *SfCHS1* and its two transcript variants, specific dsRNAs targeting *SfCHS1*, *SfCHS1a*, and *SfCHS1b* were synthesized and injected into fifth-instar nymphs. When fifth-instar nymphs on day 1 were injected with *SfCHS1* dsRNA, qPCR result showed that RNAi of *SfCHS1* strongly suppressed the expression of *SfCHS1*, thus new cuticle could not form normally due to the reduction of chitin. This result was supported by a similar study from *T*. *castaneum*^29^. In this species, *TcCHS1*-specific RNAi reduced the chitin content of whole larvae. Indeed, the morphological observation indicated that all treated planthoppers were unable to shed their old cuticle and died before reaching the adult stage. Such altered phenotypes are similar to those of *B*. *dorsalis*^23^, *Leptinotarsa decemlineata*^61^ and *L*. *migratoria*^62^ whose *CHS1* and/or *UDP-N-acetylglucosamine pyrophosphorylases* (*UAP*), two important components in chitin biosynthesis pathway, were silenced by RNAi. Further, in *L*. *migratoria*, knockdown of *LmUAP1* or *LmCHS1* led to synthesize the very thin new cuticle during their molting^62^. These results suggest once again that *UAPs* and *CHSs* play crucial role during insect ecdysis and metamorphosis.

When *CHS1a* and *CHS1b* dsRNA of the two alternative variants was injected into fifth-instar nymphs, respectively, qPCR showed no cross-silencing between *SfCHS1a* and *SfCHS1b*. *SfCHS1a* dsRNA-mediated silencing affected the growth and development of treated insects, leading to lethal phenotypes. In contrast, dsRNA-mediated silencing of *SfCHS1b* caused no obviously phenotypic defects, although the mortality was slightly increased compared with the ds*GFP*-injected control group. Our result suggested that *SfCHS1a* was essential for insect molting and metamorphosis. Similar results have been observed in *N*. *lugens*^19^ and *B*. *dorsalis*^23^, in which silencing of *CHS1a* expression by *in vivo* RNAi caused phenotypic defects in molting and resulted in mortality of the injected insects, whereas nymphs also injected with *CHS1b* dsRNA exhibited a normal phenotype. However, in *L*. *migratoria*, nymphs injected with *CHS1b* dsRNA exhibited a crimpled cuticle phenotype, resulting in over 50% mortality^16^. These results indicate that there is considerable variation in the efficiency of RNAi-mediated silencing of *CHS1b* in various insect orders.

*S*. *furcifera* is an important insect pests on rice in some Asia-Pacific countries. In recent years, destructive outbreaks of *S*. *furcifera* have been increasing in China, causing severe losses in rice yield. At present, control of planthoppers still relies upon spraying chemical insecticides. However, considering the adverse impact of insecticides on the ecological environment and on human health, new pest management strategies urgently need to be developed. A previous study demonstrated that feeding with the *trehalose phosphate synthase* (*TPS*) dsRNA in *N*. *lugens* led to reduction levels of *TPS* mRNA and disturbed the development of nymphs, suggesting that administering dsRNA corresponding to important genes by oral delivery may be a means for the control of phloem-sucking insects^39^. In another study, when *N*. *lugens* nymphs were fed on the transgenic rice plants expressing dsRNAs of the hexose transporter gene, the carboxypeptedase gene and the trypsin-like serine protease gene, levels of expression of the target genes in the midgut were suppressed; nevertheless, lethal phenotypic effects after dsRNA feeding were not observed^40^, either because the amount of dsRNA-uptake by the insects was insufficient or because RNAi target genes were not sensitive in this species. Therefore, there is an urgent need to elucidate the physiological functions of vital candidate genes from different insect species. Overall, our results indicated that injecting dsRNA of *CHS1* into *S*. *furcifera* nymphs could lead to a significant mortality, suggesting that *SfCHS1* may be a candidate gene for use in *S*. *furcifera* control.

## Conclusion

In conclusion, we successfully cloned and characterized two alternative splicing variants of the chitin synthase 1 gene (*SfCHS1*) from *S*. *furcifera*. Phylogenetic analysis demonstrated that these genes belong to the *CHS1* gene family. The genes were expressed at all developmental stages. Further, *SfCHS1* and *SfCHS1a* were mainly expressed in the integument, whereas *SfCHS1b* was predominately expressed in the gut and fat body. Our RNAi-based gene silencing inhibited the transcript levels of the corresponding variants, resulted in malformed phenotypes, and killed most of the treated nymphs. These results indicate that *SfCHS1* may be a potential target gene for RNAi-based *S*. *furcifera* control.

## Materials and Methods

### Insect rearing

The planthoppers used in the present study were originally collected from a rice paddy field in Huaxi District, Guiyang City, Guizhou Province, China. Insects were reared in the laboratory of Guizhou University on the susceptible rice variety Taichung Native-1 (TN1) under controlled conditions of temperature 25 ± 2 °C, 70 ± 10% relative humidity (RH), and a 16 h:8 h (L:D) photoperiod. The developmental stages were synchronized at each egg incubation.

### RNA extraction and cDNA cloning of *SfCHS1*

Total RNA was extracted from the whole body of fifth-instar nymphs of *S*. *furcifera* using TRIzol reagent (Invitrogen, Carlsbad, CA, USA). The integrity of total RNA was examined by 1% agarose gel electrophoresis, and a Nanodrop 2000 spectrophotometer (Thermo Fisher Scientific, Wilmington, DE, USA) was used to determine RNA concentration and purity. First-strand cDNA was synthesized from total RNA using an AMV First Strand cDNA Synthesis Kit (Sangon Biotech, Shanghai, China) with an oligodT primer, according to the user manual provided by the manufacturer.

On the basis of the transcriptome sequencing data (SRR116252) of *S*. *furcifera*^63^, four short cDNA sequences encoding *SfCHS1* were identified. To obtain a larger cDNA fragment, six pairs of gene-specific primers (Table 1) were designed using Primer Premier 6.0 (Palo Alto, CA, USA). The ends were amplified by 3′- and 5′-RACE using a SMARTer RACE Kit following the manufacturer’s instructions (Clontech, Mountain View, CA, USA). PCR amplifications were carried out using LA Taq^®^ polymerase (TaKaRa, Dalian, China) in 25-μL reaction mixtures containing 2 μL dNTP (2.5 mM), 2.5 μL 10 × LA PCR Buffer (Mg^2+^ plus), 1 μL each primer (10 mM), and l μL cDNA templates. The thermal cycling conditions were as follows: one cycle of pre-denaturation at 94 °C for 3 min, followed by 30 cycles of denaturation at 94 °C for 30 s, annealing at 50–55 °C (according to primer annealing temperature) for 30 s, and extension at 72 °C for 1–2 min (according to amplified fragment size), with a final extension at 72 °C for 10 min. The amplified products were examined by 1% agarose gel electrophoresis, and the target band of products was purified using an EasyPure^®^ Quick Gel Extraction Kit (Transgen Biotech, Beijing, China). Purified DNA was cloned into a pMD18-T vector (TaKaRa, Dalian, China) and sequenced by Sangon Biotech (Shanghai, China).

**Table 1:** Primers used for cloning the full-length cDNA of *SfCHS1* and two alternative splicing variants from *S*. *furcifera*. F: forward primer; R: reverse primer.

cDNA fragment	Primer name	Primer sequence (5′-3′)	PCR product (bp)
PCR1	SfCHS1-F1	TCTCCGACCCCATCTGTT	414
	SfCHS1-R1	GCTATCACCAGACACCAT	
PCR2	SfCHS1-F2	ACACGCTACTTCACTTATCT	870
	SfCHS1-R2	CTTCAACATCTCCATCATCTC	
PCR3	SfCHS1-F3	GCACGAGACCAACATTAGG	1193
	SfCHS1-R3	AGAGAATGAGCAGCAGGT	
PCR4	SfCHS1-F4	CTGGATTGAAGACCGTGAT	1003
	SfCHS1-R4	GCTGTTACTCGTCCGTTC	
5′ RACE	5′ RACE-R	TTGACGGTGAACTCCAGA	495
3′ RACE	3′ RACE-F1	TCCACGCATATCCAACGCCG	1566
	3′ RACE-F2	GAACGGACGAGTAACAGC	
	UPM	CTAATACGACTCACTATAGGGCAAGCAGTGGTATCAACGCAGAGT	—
	NUP	CTAATACGACTCACTATAGGGC	—

### Identification of alternative splicing exons of *SfCHS1*

It is known that the insect *CHS1* gene exists as two alternative splicing variants. To identify the alternatively spliced exons of *SfCHS1*, one pair of gene-specific primers (ASV-F: 5′-TGACGATAACAGTGATACCA-3′ and ASV-R: 5′-GAATCGGCGTCATAGTCC-3′) were designed based on the full-length sequence of *SfCHS1*. cDNA was synthesized as described above. PCR was carried out via one cycle of pre-denaturation at 94 °C for 3 min, followed by 30 cycles of denaturation at 94 °C for 30 s, annealing at 51 °C for 30 s, and extension at 72 °C for 1 min, with a final extension at 72 °C for 10 min. A 648-bp amplified product was cloned into a pMD18-T vector and sequenced.

### cDNA and amino acid sequence analysis

The sequenced fragments were assembled using SeqMan software to obtain the full-length sequence of *SfCHS1* cDNA. The nucleotide sequence was edited using DNAMAN 7.0 (Lynnon Biosoft, CA, USA). Homology searches were performed using the NCBI BLAST program (https://blast.ncbi.nlm.nih.gov/Blast.cgi). The open reading frame (ORF) of *SfCHS1* cDNA was identified using ORF finder (https://www.ncbi.nlm.nih.gov/orffinder/). The ProtParam tool at ExPASy (https://www.expasy.org/) was used to compute the molecular weight and theoretical isoelectric point (pI) of the deduced protein sequence^64^. *N*-glycosylation sites were analyzed using the NetNGlyc 1.0 Server (http://www.cbs.dtu.dk/services/NetNGlyc/), and the signal peptide was predicted using the SignalP 4.1 Server (http://www.cbs.dtu.dk/services/SignalP/). The TMHMM v.2.0 program (http://www.cbs.dtu.dk/services/TMHMM/) was used to analyze the transmembrane helices^65^. The putative coiled-coil regions were predicted using the Paircoil program^66^.

### Phylogenetic analysis of insect chitin synthases

Phylogenetic trees were constructed using MEGA 6.06 based on the neighbor-joining (NJ) method^67^. Bootstrap analyses of 1000 replications were carried out. For Phylogenetic analysis, chitin synthases were included from *Anasa tristis* (At), *Aphis glycines* (Ag), *Laodelphax striatellus* (Ls), *Nilaparvata lugens* (Nl), *Bombyx mori* (Bm), *Choristoneura fumiferana* (Cf), *Cnaphalocrocis medinalis* (Cm), *Ectropis obliqua* (Eo), *Helicoverpa armigera* (Ha), *Mamestra brassicae* (Mb), *Mamestra configurata* (Mc), *Manduca sexta* (Ms), *Ostrinia furnacalis* (Of), *Phthorimaea operculella* (Po), *Plutella xylostella* (Px), *Spodoptera exigua* (Se), *Spodoptera frugiperda* (Sfr), *Apis mellifera* (Am), *Pediculus humanus corporis* (Ph), *Anthonomus grandis* (Agr), *Tribolium castaneum* (Tc), *Anopheles gambiae* (Aga), *Anopheles quadrimaculatus* (Aq), *Bactrocera dorsalis* (Bd), *Culex quinquefasciatus* (Cq), *Drosophila melanogaster* (Dm), *Lucilia cuprina* (Lc), *Locusta migratoria manilensis* (Lm). GenBank accession numbers are as follows: AtCHS (AFM38193), AgCHS1 (AFJ00066), LsCHS1a (AFC61179), LsCHS1b (AFC61178), NlCHS1a (AFC61181), NlCHS1b (AFC61180), BmCHS (AFB83705), CfCHS1 (ACD84882), CmCHS1 (AJG44538), CmCHS2 (AJG44539), EoCHS1a (ACA50098), EoCHS1b (ACD10533), HaCHS1 (AKZ08594), HaCHS2 (AKZ08595), MbCHS1 (ABX56676), McCHS2 (AJF93428), MsCHS1 (AAL38051), MsCHS2 (AAX20091), OfCHS1 (ACB13821), OfCHS2 (ABB97082), PoCHS1 (AOE23678), PoCHS2 (AIJ50381), PxCHS1 (BAF47974), SeCHS1 (AAZ03545), SeCHS2 (ABI96087),SfrCHS2 (AAS12599), AmCHS1 (XP_395677.4), AmCHS2 (XP_001121152.2), PhCHS2 (XP_002423604), AgrCHS1 (AHY28559), AgrCHS2 (AHY28560), TcCHS1a (AAQ55059), TcCHS1b (AAQ55060), TcCHS2 (AAQ55061), AgaCHS1a (XP_321336.5), AgaCHS1b (XP_321336.4), AgaCHS2 (XP_321951), AqCHS1 (ABD74441), BdCHS1a (AEN03040), BdCHS1b (AGB51153), BdCHS2 (AGC38392), CqCHS1 (XP_001866798), CqCHS2 (XP_001864594), DmCHS1 (NP_524233), DmCHS2 (NP_524209), LcCHS1 (AAG09712), LmCHS1a (ACY38588), LmCHS1b (ACY38589), and LmCHS2 (AFK08615).

### Developmental- and tissue-specific expression of *SfCHS1* and its two alternative splicing variants

*S*. *furcifera* at stages ranging from eggs to adults were sampled to determine the developmental stage expression profiles by quantitative real-time PCR (qPCR). Five different tissue samples from the integument, fat body, gut, ovary, and head were dissected from first-day fifth-instar nymphs and third-day adults to examine tissue-specific expression. Three biological replications were performed for each sample. Total RNA was isolated from the whole body of nymphs and adults at each stage or from the different tissues using an HP Total RNA Kit (with gDNA removal columns; Omega bio-tek, Norcross, GA, USA). An AMV RT reagent Kit (Sangon Biotech) with an oligodT primer was used to synthesize first-strand cDNA. The most unique nucleotide regions of *SfCHS1*, *SfCHS1a*, and *SfCHS1b* were selected for expression analysis (the selected regions are shown in Figs 1 and 2), and the primers used for qPCR are listed in Table 2. The qPCR was performed in a CFX-96 real-time qPCR system (Bio-Rad, Hercules, CA, USA) with 20-μL reaction systems containing 10 μL FastStart Essential DNA Green Master (Roche Diagnostics, Shanghai, China), 1 μL cDNA (0.8 ng/μL), 1 μL (10 mM) of each primer, and 7 μL RNase-free water. Amplification conditions were as follows: an initial denaturation of 95 °C for 10 min and then 40 cycles of 95 °C for 30 s and 55 °C for 30 s. After the reaction, a melting-curve analysis from 65 to 95 °C was performed to confirm the specificity of the PCR. The data were normalized to the stable reference gene *18S ribosome RNA* (GenBank accession no. HM017250) based on our previous evaluations^68^. The relative expression levels were calculated using the 2^−ΔΔCt^ method^69^.

**Table 2:** Primers used for qPCR analysis and dsRNA synthesis of *SfCHS1* and its two alternative splicing variants.

Experiments	Gene name	Primer name	Primer sequence (5′-3′)	PCR product (bp)
qPCR analysis	*SfCHS1*	qCHS1-F	GATTGGTCATTGGCTTCAGA	151
		qCHS1-R	GTAATGTCTTGCTTCGTCAG	
	*SfCHS1a*	qCHS1a-F	CTTCGGTGTTTGGTTTCTT	136
		qCHS1a-R	TGGGTAACATCATCATAGGA	
	*SfCHS1b*	qCHS1b-F	GAGAAGGCGAGAATAGCA	103
		qCHS1b-R	GCAGCAAGAACACGATTA	
	18S *RNA*	q18S-F	CGGAAGGATTGACAGATTGAT	151
		q18S-R	CACGATTGCTGATACCACATAC	
dsRNA synthesis	*SfCHS1*	dsCHS1-F	TAATACGACTCACTATAGGG CTGACGAAGCAAGACATTAC	491
		dsCHS1-R	TAATACGACTCACTATAGGG CACTATCACAGCCATCATTATC	
	*SfCHS1a*	dsCHS1a-F	TAATACGACTCACTATAGGG GAATAGCGTCGGATCTCA	173
		dsCHS1a-R	TAATACGACTCACTATAGGG CTCTTGGGTAACATCATCAT	
	*SfCHS1b*	dsCHS1b-F	TAATACGACTCACTATAGGG GAGAAGGCGAGAATAGCA	170
		dsCHS1b-R	TAATACGACTCACTATAGGG TCGACGTAAGTGATATTGG	
	*GFP*	dsGFP-F	TAATACGACTCACTATAGGG AAGGGCGAGGAGCTGTTCACCG	707
		dsGFP-R	TAATACGACTCACTATAGGG CAGCAGGACCATGTGATCGCGC	

### Functional analysis of *SfCHS1* and its two alternative splicing variants using RNAi

To further investigate the biological functions of *SfCHS1* and its two alternative splicing variants, *SfCHS1a* and *SfCHS1b*, RNAi was carried out by injecting *S*. *furcifera* nymphs with sequence-specific dsRNA. The most unique nucleotide regions of *SfCHS1*, *SfCHS1a* and *SfCHS1b* were selected for dsRNA synthesis (the synthesized regions are shown in Figs 1 and 2), and the primers added a T7 RNA polymerase promoter (Table 2) were used to synthesize dsRNA. Templates for *in vitro* transcription reactions were synthesized by PCR from the plasmid DNA of *SfCHS1*, *SfCHS1a*, and *SfCHS1b* using primers. The PCR products of *SfCHS1*, *SfCHS1a*, and *SfCHS1b* were subcloned and sequenced to determine the specificity. The expected fragments were then purified using an EasyPure^®^ Quick Gel Extraction Kit (Transgen Biotech). The concentration of the purified products was determined using a Nanodrop 2000 spectrophotometer (Thermo Fisher Scientific) and these products were then used for *in vitro* transcription reactions.

dsRNAs were synthesized using a MEGAscript^®^ RNAi Kit (Ambion, Carlsbad, CA, USA) according to the user manual provided by the manufacturer. *In vivo* RNAi in *S*. *furcifera* nymphs was carried out as previously described^19,70^. First-day fifth-instar nymphs were anesthetized with carbon dioxide for approximately 30 s and subsequently used for microinjection. Each group included 50 nymphs and treatments were performed in triplicate. One hundred nanograms of dsRNA was injected into nymphs between the prothorax and mesothorax using a Nanoliter 2010 Injector (injection speed, 25 nL/s) (World Precision Instruments, FL, USA). Equivalent volumes of ds*GFP* were used for control injections. Injected nymphs were maintained on fresh rice under the conditions described above until eclosion, and thereafter phenotype and mortality were observed daily. Photographs were taken using a Keyence VH-Z20R stereoscopic microscope (Keyence, Osaka, Japan). Subsequent to injection, 10 nymphs were selected randomly from each replication for mRNA-level detection.

### Statistical analysis

Statistical analysis of all data was performed using SPSS 13.0 software (IBM Inc., Chicago, IL, USA). Data values are represented as the mean ± *SE* of three replications. A one-way ANOVA and Duncan’s multiple range test (*P* < 0.05) were used to calculate the relative expression of each sample. For RNAi experiments, significant differences in mRNA levels between each of the ds*RNA*-injected groups and the ds*GFP* group were analyzed using *t*-tests.

## Data Availability

The data were deposited in GenBank with accession numbers KY350143 (*SfCHS1a*) and KY350144 (*SfCHS1b*).

## References

[1] Merzendorfer H, Zimoch L (2003). Chitin metabolism in insects: structure, function and regulation of chitin synthases and chitinases. J. Exp. Biol..

[2] Merzendorfer H (2006). Insect chitin synthases: a review. J. Comp. Physiol. B.

[3] Daimon T (2003). A *Bombyx mori* gene, BmChi-h, encodes a protein homologous to bacterial and baculovirus chitinases. Insect Biochem. Mol. Biol..

[4] Minamoto T (2015). Saccharification of beta-chitin from squid pen by a fermentation method using recombinant chitinase-secreting *Escherichia coli*. Appl. Biochem. Biotechnol..

[5] Tang WJ, Fernandez JG, Sohn JJ, Amemiya CT (2015). Chitin is endogenously produced in vertebrates. Curr. Biol..

[6] Zhu KY, Merzendorfer H, Zhang WQ, Zhang JZ, Muthukrishnan S (2016). Biosynthesis, turnover, and functions of chitin in insects. Annu. Rev. Entomol..

[7] Hegedus D, Erlandson M, Gillott C, Toprak U (2009). New insights into peritrophic matrix synthesis, architecture, and function. Annu. Rev. Entomol..

[8] Moussian B (2010). Recent advances in understanding mechanisms of insect cuticle differentiation. Insect Biochem. Mol. Biol..

[9] Arakane Y, Taira T, Ohnuma T, Fukamizo T (2012). Chitin-related enzymes in agro-biosciences. Curr. Drug Targets.

[10] Alvarenga ESL (2016). Chitin is a component of the *Rhodnius prolixus* midgut. Insect Biochem. Mol. Biol..

[11] Shi JF, Mu LL, Chen X, Guo WC, Li GQ (2016). RNA interference of chitin synthase genes inhibits chitin biosynthesis and affects larval performance in *Leptinotarsa decemlineata* (Say). Int. J. Biol. Sci..

[12] Macedo LLP (2017). Knocking down chitin synthase 2 by RNAi is lethal to the cotton boll weevil. Biotechnol. Res. Innovat..

[13] Ampasala DR (2011). An epidermis-specific chitin synthase cDNA in *Choristoneura fumiferana*: cloning, characterization, developmental and hormonal-regulated expression. Arch. Insect Biochem..

[14] Qu MB, Yang Q (2011). A novel alternative splicing site of class A chitin synthase from the insect *Ostrinia furnacalis* - gene organization, expression pattern and physiological significance. Insect Biolchem. Mol. Biol..

[15] Zhuo WW (2014). Chitin synthase A: a novel epidermal development regulation gene in the larvae of *Bombyx mori*. Mol. Biol. Rep..

[16] Zhang JZ (2010). Silencing of two alternative splicing-derived mRNA variants of chitin synthase 1 gene by RNAi is lethal to the oriental migratory locust, *Locusta migratoria manilensis* (Meyen). Insect Biochem. Mol. Biol..

[17] Liu XJ (2012). Characterization of a midgut-specific chitin synthase gene (*LmCHS2*) responsible for biosynthesis of chitin of peritrophic matrix in *Locusta migratoria*. Insect Biochem. Mol. Biol..

[18] Bansal R, Mian MA, Mittapalli O, Michel AP (2012). Characterization of a chitin synthase encoding gene and effect of diflubenzuron in soybean aphid. Aphis glycines. Int. J. Biol. Sci..

[19] Wang Y (2012). Chitin synthase 1 gene and its two alternative splicing variants from two sap-sucking insects, *Nilaparvata lugens* and *Laodelphax striatellus* (Hemiptera: Delphacidae). Insect Biochem. Mol. Biol..

[20] Shang F (2016). Identification, characterization and functional analysis of chitin synthase gene in the brown citrus aphid, *Toxoptera citricida* (Hemiptera, Aphididae). Insect Mol. Biol..

[21] Zhang X, Zhang JZ, Park Y, Zhu KY (2012). Identification and characterization of two chitin synthase genes in African malaria mosquito. Anopheles gambiae. Insect Biochem. Mol. Biol..

[22] Chen L, Yang WJ, Cong L, Xu KK, Wang JJ (2013). Molecular cloning, characterization and mRNA expression of a chitin synthase 2 gene from the oriental fruit fly, *Bactrocera dorsalis* (Diptera: Tephritidae). Int. J. Mol. Sci..

[23] Yang WJ, Xu KK, Cong L, Wang JJ (2013). Identification, mRNA expression, and functional analysis of chitin synthase 1 gene and its two alternative splicing variants in oriental fruit fly. Bactrocera dorsalis. Int. J. Biol. Sci..

[24] Merzendorfer H (2011). The cellular basis of chitin synthesis in fungi and insects: Common principles and differences. Eur. J. Cell Biol..

[25] Zimoch L, Merzendorfer H (2002). Immunolocalization of chitin synthase in the *Tobacco hornworm*. Cell Tissue Res..

[26] Silva CP (2004). Occurrence of midgut perimicrovillar membranes in paraneopteran insect orders with comments on their function and evolutionary significance. Arthropod Struct. Dev..

[27] Albuquerque-Cunha JM (2009). Cytochemical characterization of microvillar and perimicrovillar membranes in the posterior midgut epithelium of *Rhodnius prolixus*. Arthropod Struct. Dev..

[28] Shirk PD (2015). Unique synteny and alternate splicing of the chitin synthases in closely related heliothine moths. Gene.

[29] Arakane Y (2005). The *Tribolium* chitin synthase genes *TcCHS1* and *TcCHS2* are specialized for synthesis of epidermal cuticle and midgut peritrophic matrix. Insect Mol. Biol..

[30] Arakane Y, Specht CA, Kramer KJ, Muthukrishnan S, Beeman RW (2008). Chitin synthases are required for survival, fecundity and egg hatch in the red flour beetle. Tribolium castaneum. Insect Biochem. Mol. Biol..

[31] Firmino AA (2013). Transcriptome analysis in cotton boll weevil (*Anthonomus grandis*) and RNA interference in insect pests. PLoS One.

[32] Tian HG (2009). Developmental control of a lepidopteran pest Spodoptera exigua by ingestion of bacteria expressing dsRNA of a non-midgut gene. PLoS One.

[33] Moussian B (2015). Deciphering the genetic programme triggering timely and spatially-regulated chitin deposition. PLoS Genet..

[34] Wang Y, Zuber R, Oehl K, Norum M, Moussian B (2015). Report on *Drosophila melanogaster* larvae without functional tracheae. J. Zool..

[35] Moussian B, Schwarz H, Bartoszewski S, Nüsslein-Volhard C (2005). Involvement of chitin in exoskeleton morphogenesis in *Drosophila melanogaster*. J. Morphol..

[36] Hu SJ (2015). Projecting distribution of the overwintering population of *Sogatella furcifera* (Hemiptera: Delphacidae), in Yunnan, China with analysis on key influencing climatic factors. J. Insect Sci..

[37] Wang Z, Zhou C, Long GY, Yang H, Jin DC (2018). Sublethal effects of buprofezin on the development, reproduction, and chitin synthase 1 gene (SfCHS1) expression in the white-backed planthopper, *Sogatella furcifer*a (Hemiptera: Delphacidae). J. Asia-Pac. Entomol..

[38] Baum JA (2007). Control of coleopteran insect pests through RNA interference. Nat. Biotechnol..

[39] Chen J (2010). Feeding-based RNA interference of a trehalose phosphate synthase gene in the brown planthopper. Nilaparvata lugens. Insect Mol. Biol..

[40] Zha WJ (2011). Knochdown of midgut genes by dsRNA-transgenic plant-mediated RNA interference in the hemipteran insect *Nilaparvata lugens*. PLoS One.

[41] Zhu YC (2002). Sequence of a cDNA and expression of the gene encoding a putative epidermal chitin synthase of *Manduca sexta*. Insect Biochem. Mol. Biol..

[42] Cohen E (2001). Chitin synthesis and inhibition: a revisit. Pest Manag. Sci..

[43] Wass MN, Kelley LA, Sternberg MJE (2010). 3DLigandSite: predicting ligand-binding sites using similar structures. Nucleic Acids Res..

[44] Rezende GL (2008). Embryonic desiccation resistance in *Aedes aegypti*: presumptive role of the chitinized serosal cuticl. BMC Del. Biol..

[45] Tellam RL, Vuocolo T, Johnson SE, Jarmey J, Pearson RD (2000). Insect chitin synthase: cDNA sequence, gene organization and expression. Eur. J. Biochem..

[46] Arakane Y (2004). Characterization of two chitin synthase genes of the red flour beetle, *Tribolium castaneum*, and alternate exon usage in one of the genes during development. Insect Biochem. Mol. Biol..

[47] Skehel JJ, Wiley DC (1998). Coiled coils in both intracellular vesicle and viral membrane fusion. Cell.

[48] Burkhard P, Stetefeld J, Strelkov SV (2001). Coiled coils: a highly versatile protein folding motif. Trends Cell Biol..

[49] Melia TJ (2002). Regulation of membrane fusion by the membrane-proximal coil of the t-SNARE during zippering of SNAREpins. J. Cell Biol..

[50] Chen XF (2007). The class A chitin synthase gene of *Spodoptera exigua*: Molecular cloning and expression patterns. Insect Biochem. Mol. Biol..

[51] Irving P, Fenton B (1996). Molecular and adaptive variation in aphid guts. Scott. Crop Res. Inst. Annu. Rep..

[52] Hogenkamp DG (2005). Chitin synthase genes in *Manduca sexta*: characterization of a gut-specific transcript and differential tissue expression of alternately spliced mRNAs during development. Insect Biochem. Mol. Biol..

[53] Ashfaq M, Sonoda S, Tsumuki H (2007). Developmental and tissue-specific expression of CHS1 from *Plutella xylostella* and its response to chlorfluazuron. Pestic. Biochem. Physiol..

[54] Zhai YF (2017). Identification and functional analysis of chitin synthase A in oriental armyworm. Mythimna separata. Proteomics.

[55] Moreira MF (2007). A chitin-like component in *Aedes aegypti* eggshells, eggs and ovaries. Insect Biochem. Mol. Biol..

[56] Zhang X, Zhang JZ, Zhu KY (2010). Chitosan/double-stranded RNA nanoparticle-mediated RNA interference to silence chitin synthase genes through larval feeding in the African malaria mosquito (*Anopheles gambiae*). Insect Mol. Biol..

[57] Jia S, Wan PJ, Zhou LT, Mu LL, Li GQ (2013). Molecular cloning and RNA interference-mediated functional characterization of a Halloween gene spook in the white-bached planthopper *Sogatella furcifera*. BMC Mol. Biol..

[58] Zhao LN (2016). Functional characterization of three trehalase genes regulating the chitin metabolism pathway in rice brown planthopper using RNA interference. Sci. Rep-UK..

[59] Chen C, Yang H, Tang B, Yang WJ, Jin DC (2017). Identification and functional analysis of chitinase 7 gene in the white-bached planthopper *Sogatella furcifera*. Comp. Biochem. Phys. B.

[60] Zhang L (2017). Glycogen phosphorylase and glycogen synthase: gene cloning and expression analysis reveal their role in trehalose metabolism in the brown planthopper, *Nilaparvata lugens* Stål (Hemiptera: Delphacidae). J. Insect Sci..

[61] Shi JF, Fu J, Mu LL, Guo WC, Li GQ (2016). Two *leptinotarsa* uridine diphosphate N-acetylglucosamine pyrophosphorylases are specialized for chitin synthesis in larval epidermal cuticle and midgut peritrophic matrix. Insect Biochem. Mol. Biol..

[62] Liu XJ (2018). Identification of *LmUAP1* as a 20-hydroxyecdysone response gene in the chitin biosynthesis pathway from the migratory locust, *Locusta migratoria*. Insect Sci..

[63] Zhou C, Yang H, Wang Z, Long GY, Jin DC (2018). Comparative transcriptome analysis of *Sogatella furcifera* (Horváth) exposed to different insecticides. Sci. Rep-UK..

[64] Gasteiger, E. *et al*. Protein identification and analysis tools on the ExPASy Server, (In)Walker, J. M. (Ed.): The proteomics protocols handbook, Humana Press. pp. 571–607 (2005).

[65] Krogh A, Larsson B, Heijne G, Sonnhammer ELL (2001). Predicting transmembrane protein topology with a hidden Markov model: application to conplete genomes. J. Mol. Biol..

[66] Berger B (1995). Predicting coiled coils by use of pairwise residue correlations. Proc. Natl. Acad. Sci..

[67] Tamura K, Stecher G, Peterson D, Filipske A, Kumar S (2013). MEGA6: Molecular evolutionary genetics analysis version 6.0. Mol. Biol. Evol..

[68] Yu JL, An ZF, Liu XD (2014). Wingless gene cloning and its role in manipulating the wing dimorphism in the white-backed planthopper, *Sogatella furcifera*. BMC Mol. Biol..

[69] Livak KJ, Schmittgen TD (2001). Analysis of relative gene expression data using realtime quantitative PCR and the 2^−ΔΔCt^ method. Methods.

[70] Liu S, Ding Z, Zhang C, Yang B, Liu Z (2010). Gene knockdown by intro-thoracic injection of double-stranded RNA in the brown planthopper, *Nilaparvata lugens*. Insect Biochem. Mol. Biol..

